# eBASIS (Bioactive Substances in Food Information Systems) and Bioactive Intakes: Major Updates of the Bioactive Compound Composition and Beneficial Bioeffects Database and the Development of a Probabilistic Model to Assess Intakes in Europe

**DOI:** 10.3390/nu9040320

**Published:** 2017-03-23

**Authors:** Jenny Plumb, Sandrine Pigat, Foteini Bompola, Maeve Cushen, Hannah Pinchen, Eric Nørby, Siân Astley, Jacqueline Lyons, Mairead Kiely, Paul Finglas

**Affiliations:** 1 Institute of Food Research, Norwich NR4 7UA, UK; hannah.pinchen@ifr.ac.uk (H.P.); paul.finglas@ifr.ac.uk (P.F.); 2 Creme Global, Grand Canal Quay, Dublin 2, Ireland; sandrine.pigat@cremeglobal.com (S.P.); foteini.bompola@cremeglobal.com (F.B.); maeve.cushen@cremeglobal.com (M.C.); 3 Polytec ApS, Niva, 2990 Copenhagen, Denmark; ein@polytec.dk; 4 EuroFIR Association Internationale Sans But Lucratif, 40, Rue Washington, 1050 Brussels, Belgium; sa@eurofir.org; 5 School of Food and Nutritional Sciences, University College Cork, T12 Y337 Cork, Ireland; jacqueline.lyons@ucc.ie (J.L.); m.kiely@ucc.ie (M.K.)

**Keywords:** eBASIS, database, health claims, bioactive compounds, composition, phytochemicals, exposure assessment

## Abstract

eBASIS (Bioactive Substances in Food Information Systems), a web-based database that contains compositional and biological effects data for bioactive compounds of plant origin, has been updated with new data on fruits and vegetables, wheat and, due to some evidence of potential beneficial effects, extended to include meat bioactives. eBASIS remains one of only a handful of comprehensive and searchable databases, with up-to-date coherent and validated scientific information on the composition of food bioactives and their putative health benefits. The database has a user-friendly, efficient, and flexible interface facilitating use by both the scientific community and food industry. Overall, eBASIS contains data for 267 foods, covering the composition of 794 bioactive compounds, from 1147 quality-evaluated peer-reviewed publications, together with information from 567 publications describing beneficial bioeffect studies carried out in humans. This paper highlights recent updates and expansion of eBASIS and the newly-developed link to a probabilistic intake model, allowing exposure assessment of dietary bioactive compounds to be estimated and modelled in human populations when used in conjunction with national food consumption data. This new tool could assist small- and medium-sized enterprises (SMEs) in the development of food product health claim dossiers for submission to the European Food Safety Authority (EFSA).

## 1. Introduction

A bioactive can be defined as any non-nutrient present in food that could exert a beneficial or toxic effect when ingested [1]. In recent years there has been increasing interest in the effects of these bioactive compounds on health and wellbeing. Plant foods have traditionally been the focus of research on bioactives but, more recently, non-plant foods are being considered rich sources of bioactive compounds, with ongoing research into the potential health effects of non-plant bioactives in meat [2,3,4] and dairy [5,6,7]. Observational and clinical studies in recent years propose that the presence of bioactive compounds can largely explain the epidemiological evidence for potential health benefits associated with increased consumption of fruit and vegetables, with studies suggesting that flavonoid-rich diets are correlated specifically with a reduced risk of chronic disorders, such as cardiovascular diseases [8,9,10], type 2 diabetes [11,12], and some cancers [13,14].

Although not widely studied, meat and meat products also contain important bioactive compounds. These include amino acids and a range of peptides derived from meat protein through enzymatic hydrolysis [15,16,17]. Concentrations of these bioactives in meat and meat products also contribute to the quality and taste of the meat products, and they appear to be affected by characteristics of the animal, its rearing and the predominant type of muscle in the cut [18,19,20]. In addition, the processing and cooking of meat and meat products can affect concentrations. It is, therefore, important to begin the process of collating both compositional data for bioactives in these products and information about their bioeffects in humans. 

There is an increasing awareness in the potential health benefits of bioactive compounds and, as such, there is considerable interest in databases, the ways to improve the estimation of intake levels [21], and methods to study the nature and dose activity relationships of their biological effects. Since there is potential for food-based health-beneficial bioactives to provide protection against diseases, the need for easily accessible information on composition, intake, and activity of these compounds is crucial for researchers; hence, the need for data provided in a convenient and widely accessible form. There are three core databases available providing extensive data on the composition of bioactive compounds in foods: the USDA flavonoid database [22]; Phenol-Explorer [23] covering polyphenols; and eBASIS (Bioactive Substances in Food Information Systems) [24], which includes both composition and beneficial bioeffects of not only polyphenols, but also of additional bioactive compound classes. The databases contain similar compositional data as they are all based on data from the literature. However, the best value is from databases most recently updated with the greatest amount of data for each food. eBASIS is unique in the inclusion of biological effects data which may be used in the preparation and evaluation of health claim dossiers [25].

The aim of this work was to extend the contents of the eBASIS database to include new compositional and biological effects data for bioactive compounds of plant origin and, uniquely, include meat bioactives, for which some evidence of a potential beneficial effect has been reported [3]. In addition, we report how the database can be linked to dietary population intakes of bioactives; information that is required in the development of the European Food Safety Authority (EFSA) health claim dossiers. This was part of wider research to develop tools and resources to study the relationships between intakes of bioactive compounds and cardiovascular health in humans by the EU-funded project BACCHUS, ‘Beneficial effects of bioactive compounds in humans’ [26]. Part of the BACCHUS project focused on assessing population bioactive intakes, case studies on the dietary impact of foods and products high in bioactives, and the development of a platform to analyse those bioactive intakes. National food consumption surveys, which are representative of dietary habits and used to assess food and nutrient intakes, as well as the health status in a population, have been used to assess the intake of bioactive compounds.

## 2. Materials and Methods

### 2.1. eBASIS

eBASIS is a comprehensive, searchable database, containing up-to-date quality evaluated scientific information from peer reviewed publications, covering the composition and beneficial bioeffects of bioactive compounds present in foods. It is a valuable resource for food regulatory and advisory bodies, researchers interested in diet and health relationships, as well as product developers within the food industry. eBASIS originated in 1998 in CD-ROM form and has, over the subsequent years, been moved online, extended, and expanded to produce EuroFIR eBASIS in 2007 [1], and the ePlantLIBRA plant food supplement database in 2015 [27]. The recently updated eBASIS documented here was funded via the BACCHUS project [26] involving 28 research institutes, universities, and small and medium-sized enterprises (SMEs) across Europe. The primary focus of BACCHUS was the relationship between consumption of polyphenols and bioactive peptides, and the potential beneficial physiological effects related to human cardiovascular health. A principal aim of the project was the development of tools and resources that would enable the generation of robust scientific evidence in this area, eBASIS being one of these tools.

**Figure 1:**
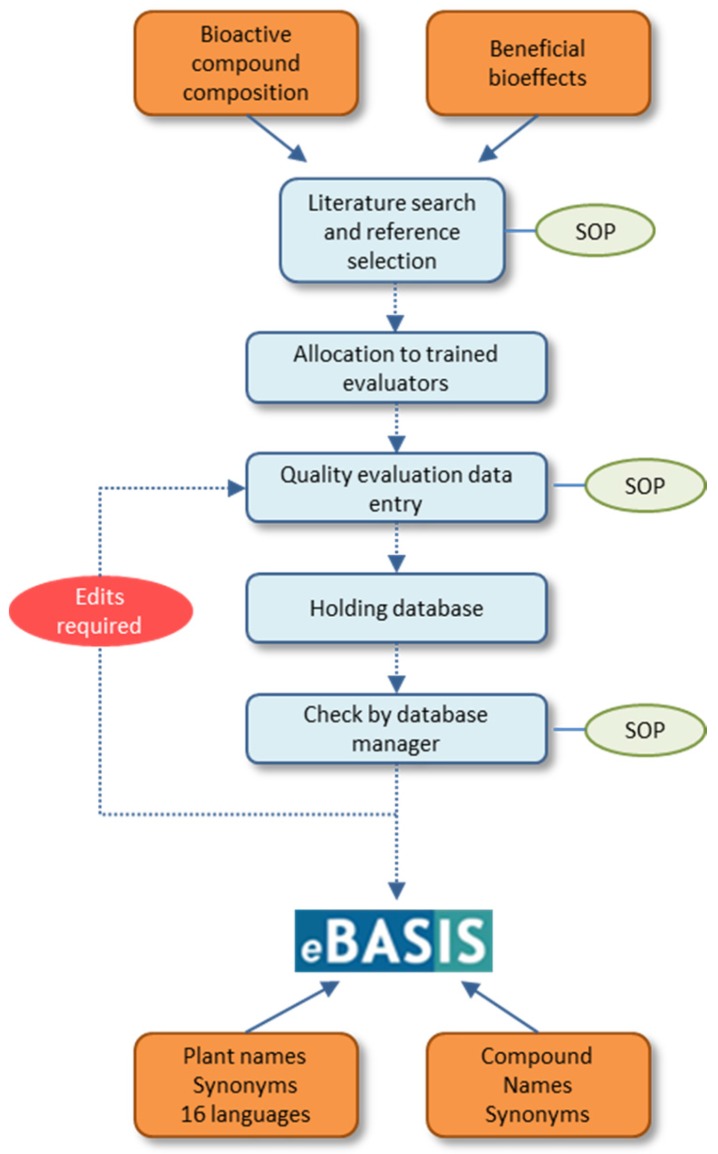
eBASIS compilation procedure, showing major steps and standard operating procedures (SOPs).

eBASIS [28], hosted by EuroFIR AISBL [29], with access via membership packages or within EU projects, is a relational database served by Microsoft Windows Server version 2008 R2 Enterprise, Microsoft Internet Information Services version 6.1, and Microsoft SQL Server version 2008. (Microsoft, Redmond, WA, USA), which is also verified to operate on version 2012. eBASIS is managed by two institutions, the composition data by the Institute of Food Research, UK and bioeffects data by University College Cork, Ireland. Data entry is carried out by trained evaluators sourced from EU-funded projects and all inputs are checked by database managers before appearing in the database. The data entry goal is to source, extract, and quality-assess data from peer reviewed publications concerned with the composition and biological effects of bioactive compounds in foods. All evaluators are fully trained in the use of the form with regular evaluator assessments to check performance and to ensure uniformity between evaluators. The data input form is designed to promote consistency in data entry with 35 fields used in composition, and 42 in bioeffects, records. The forms are designed to be unambiguous and simple to use and come with clear instructions for use. Where possible, pick lists are used, which also enable detailed searches to be carried out by database users; free text fields are intentionally limited to simplify reporting and data analysis of eBASIS contents. All inputs are submitted to a holding database for inspection by a database manager who inspects all fields in the form, checking for inconsistencies or errors before accepting into eBASIS. Publications are sourced and assessed through standardised search and selection protocols, and each food-compound combination is entered separately. Standard operating procedures (SOPs) are followed in each step of the compilation process. The compilation procedure with critical steps, SOPs and database composition is summarised in Figure 1.

Data are searched for and retrieved via three reporting systems: compositional data plants; compositional data meats; and beneficial bioeffects data. Searching is user-led through a variety of parameters: individual compounds, food, or a combination of both compound and food. The level of detail viewed is also user-led; default fields are provided with the option to include additional fields, such as reference, analytical, or sampling information. The report, once produced, can be downloaded and manipulated in spreadsheets, and the list of references used to compile the individual report can be viewed and saved for inputting into reference library software. Search criteria can also be saved for further use.

### 2.2. eBASIS Updates

A major portion of the work to expand eBASIS was to increase the quantity of evaluated data for the composition and biological effects of bioactive compounds in fruits, vegetables, and meats from peer-reviewed publications (see Section 2.3 for detail). These included updates on the current contents and new plant/compounds added where there were deemed to be gaps in the database. Since the database was originally developed for plant bioactives it has been enhanced to allow the addition of peer reviewed and new analytical data on bioactives in meat (detailed in Section 2.2.1). eBASIS querying and output systems have also been revised and improved.

Although the majority of new data in eBASIS stems from published literature, a quantity of new analytical data produced for the EU BACCHUS project has been entered, prior to peer reviewed publication, adhering to identical quality assessment systems (see Section 2.3).

#### 2.2.1. Database Revisions: Procedure for Addition of Meat Bioactive Data

The database expansion involved revision of the structure to accommodate new data, specifically peptides in meat and meat products. Originally, eBASIS was designed for plant material data and related compounds and, therefore, some structural changes were required to adapt the database to this new information whilst maintaining an appropriate structure and functionality for plant data.

eBASIS uses online forms to enter quality evaluated data using a systematic approach, described by Gry et al. [1]. Integral to the design of the forms is the category of data to be extracted, i.e., numerical, fixed text (such as yes/no), pick lists and free text fields. The revised fields required for entry of meat composition data are detailed in Supplementary Table S1.

The processing section of the input form is particularly important for processed meat products, such as ham, sausages, etc. Within eBASIS, processing choice is based on LanguaL [28] processing codes; multiple selections can be made by the evaluator when selecting processing methods. The existing eBASIS processing options were adapted to include new categories in order to create additional definitions for meat product processing. As an example, the single term “cured” applies to all meat products treated with nitrite (this also includes bacon, cooked ham, and cooked sausages), while a new term dry-cured (or simply dry) refers to typical Spanish hams or Parma hams, etc., where drying is applied and nitrificants are optional (but not cooking). Additional processing methods for inclusion in eBASIS, particularly relevant for meat products, included: cured (alone), dry-cured, fermented, and dry-fermented.

A review of the literature on the composition of meats, such as papers by Aristoy et al. [18], Cornet et al. [15], Mora et al. [20], and Peiretti et al. [30], indicated that sampling methods were unlikely to be described in as much detail as many of the plant bioactive composition papers. However, where information is present, the sampling fields should be completed: sample plan; sample handling; sample year; primary sample unit size; primary sample units; analytical sample size; analytical portion size; analytical portions; and portion replicates.

The information collected in the compositional data section largely remains the same, with only the addition of new meat bioactive peptide compound classes and compounds, such as anserine, carnosine, citrulline, and taurine.

The purpose of the Quality Assessment Section remains the same [1], with peer-reviewed data included in eBASIS critically evaluated across a number of key areas. Definitions of the seven areas covered by this evaluation have been adapted slightly to assure relevance for meat and meat products: food description; processing; sampling plan; sample handling; compound identification; analytical method; and analytical performance.

### 2.3. Addition of New Data: Plants and Meats

New data on composition and bioeffects have been added, and these include quality-evaluated data from peer-reviewed publications and new analytical data produced as part of the BACCHUS project [26]. New compositional data generated within BACCHUS was entered into the database prior to peer-reviewed publication and, as such, it has been necessary to highlight that some data are unpublished. All unpublished data have a unique leading letter in the reference code to identify them as unpublished. The quality systems used for data entry are identical for all data. If data are published subsequently, the unpublished data entries will be replaced with full citations.

New analytical data carried out for this project consists of: HPLC analysis of ellagitannins and ellagic acid conjugates in strawberry, raspberry, cloudberry, blackberry, and pomegranate from Norway, Finland, Turkey and Spain; the natural dipeptides carnosine and anserine quantified in 24 dry-cured hams; and nsLTP2 [31] in 60 accessions of wheat genotypes. Full documentation of the sampling, extraction, and analytical determinations were included within eBASIS prior to peer reviewed publication in 2017.

For new data from peer reviewed publications, a priority list of foods/compounds for both composition and bioeffects areas was developed to enhance the database; these included updates on the current contents and new plant/compounds added where there were deemed to be gaps in the database. Aronia (chokeberry) and *Rubus chamaemorus* (cloudberry) have been added to eBASIS for the first time as part of this task, with orange, apple, pomegranate, blackberry, strawberry, raspberry, and walnut selected for major updates including all bioactive compounds present. The literature was searched comprehensively on a plant-by-plant basis for relevant publications using combinations of terms related to the plant (or food) plus compound classes and individual compounds of interest.

Searches were undertaken using the electronic databases available at each leading institution. Web of Science [32] was used as the main search database for compositional data and Web of Science, MEDLINE, and CAB Abstracts used for biological effects. These literature searches remained largely the same as documented in the original eBASIS 2007 publication [1]. For compositional data, searches were designed using three groups of search terms: plant or food terms; compound or class terms; and composition or analysis terms. These were combined within each area using the OR Boolean operator, and the three areas combined with the AND Boolean operator. Therefore, literature would only be returned by the search engine if it contained one of the designated plant/food terms AND one of the designated compound terms AND one of the designated composition terms. Wildcards (e.g., *) were used at the start and/or end of words or partial words to pick up additional variations that shared common trunks—this was particularly useful for related compounds, e.g., *catechin* would pick up epicatechin, catechins, etc. The genus and species names of plants were used where appropriate, and alternative common or scientific names for plants were researched. Consideration was given to variations in spelling and endings (using wildcard operators as appropriate). Composition terms were more difficult to determine as they include words used frequently in scientific reporting (e.g., content, composition, analysis) and there is no standardized reporting terminology for composition papers. However, papers not mentioning any of the selected composition terms would be highly unlikely to be relevant to the database.

**Figure 2:**
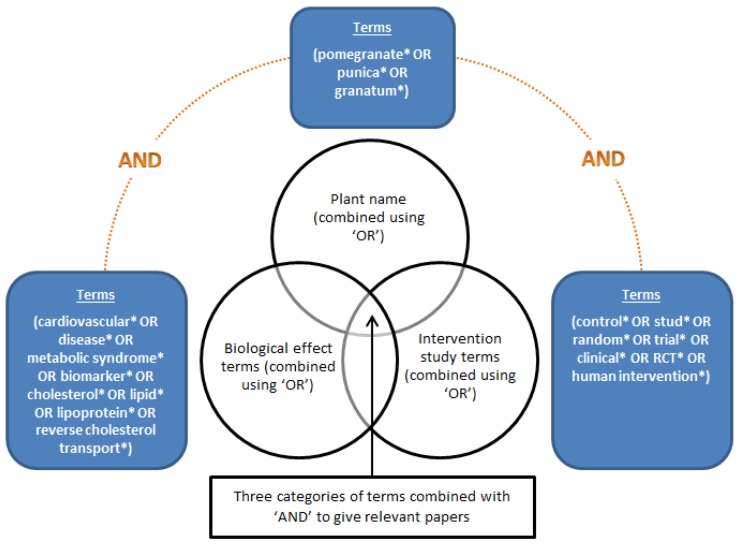
An example of search design for beneficial bioeffects in pomegranate.

The bioeffects searches were designed using three groups of search terms: prioritised biological effect search terms, e.g., cardiovascular disease, hypertension, oxidative stress, metabolic syndrome; plant name (Latin name and common name); human intervention study terms, e.g., control*, stud*, random*, trial*, clinical*, RCT, human intervention*. The literature reviews carried out on beneficial data remain largely the same as that used in the development of the ePlantLIBRA database, a compositional and biological activity database for bioactive compounds in plant food supplements [33]. An example of search design for beneficial bioeffects in pomegranate is shown in Figure 2. After searching for suitable peer-reviewed publications, the resulting literature was reviewed and some papers were excluded, examples for reasons for exclusion are as follows; Data on compounds not prioritised in eBASIS database; Non-edible plant parts; development of analytical methods for identification only, no analytical data; unacceptable analytical procedures; review articles; no control included in trial (non-RCT); unacceptable experimental procedures; insufficient documentation for evaluation.

### 2.4. Linking of eBASIS Data to Probabilistic Intake Model to Assess Bioactive Intakes

To create an intake model for BACCHUS, food consumption data from national food consumption surveys from the UK, the Netherlands, and Spain were used: United Kingdom [34] National Diet and Nutrition Survey: Rolling Programme 2008–2012 Adults (NDNS Adults) (N 1 = 2083) (Age 18+, 4 day un-weighed food diary)); Ireland [35] National Adult Nutrition Survey 2011 (NANS) (N = 1500) (Age 18+, four-day weighed food diary); and Spain [36] (2009–2010 (ENIDE) (N = 3386) (Age 18+, 3 days dietary record) . In all cases N indicates the number of subjects included in each survey (total population).These were linked to bioactive compositional data, which was extracted from eBASIS.

Data exported from eBASIS were formatted into compounds per food, as consumed in the food consumption data, before being mapped to foods consumed in the intake surveys. To exclude data that were not applicable to foods as consumed, further refinements were carried out on the eBASIS data before merging them with the consumption surveys e.g., removal of *peel only* data and data expressed as dry weight. EuroFIR food classification, heat treatment, cooking method, treatment applied, and preservation method were considered to distinguish between data for raw foods and for foods that have undergone some processing.

Within the three food consumption surveys, the consumed foods, where applicable, were grouped into the plant names listed in Table 1. Composite foods were broken down into eBASIS food components using recipes, such as the NANS Recipe Database [35], and an average taken from standard online recipes. The plant component was defined as a fraction of the total amount of the dish consumed (in weight) before being assigned to the new food group. The bioactive composition data was multiplied by the fraction in order to assess the bioactive intake from the respective amount consumed of the plant component of the food. The bioactive data was linked to the foods consumed via a composition table. This table linked the food codes from each of the three surveys to the formatted eBASIS concentration data via matching the plant name from eBASIS to the foods consumed in the surveys. Table 1 lists the compound classes analysed in this study, the food/plant names, and other selected output fields that were exported.

For each food-compound pair, a discrete data distribution of bioactive concentrations was created, allowing for probabilistic sampling of eBASIS concentration data for a given plant. As a result, the intake model uses multiple concentration records for each food-compound combination. The Creme Nutrition^®^ model was used to assess food bioactive intake distributions [37]. Daily bioactive intakes from each food were quantified by using the amount of food consumed (g) and the bioactive concentration record (mg per kg) taken from the distribution for each reported day of consumption for each subject in the three surveys. Weighted mean daily and percentile intakes for the total population were generated. In order to validate the results, composition and intake data were compared to existing databases [23,38] and intake studies [39,40].

### 2.5. Case Study: Scenario Modelling to Predict Dietary Intake of Epicatechin

Bioactive compositional data from specific products used in human intervention studies carried out within BACCHUS were provided to model intakes in the UK population and predict epicatechin intakes based on different scenarios. For this case study doses used within the human trials were applied to simulate the impact of apple polyphenol extract capsules within the diet. Dietary intakes of epicatechin, after incorporating the capsules at two different doses into the diet, were assessed: epicatechin at 70 mg/capsule and epicatechin at 140 mg/capsule. Dietary supplement consumers within the NDNS survey, defined as consuming a food from the group Dietary Supplements for at least one eating occasion of the four-day survey, were given new eating events comprised of one capsule per day at the two doses above. Probabilities of a supplement consumer to consume the above product were also applied at 0.5, 0.75, and 1.0, meaning that supplement consumers were likely to take an epicatechin capsule with a 50%, 75%, and 100% chance.

### 2.6. eBASIS-Creme Global Exposure Interface

Baseline results from all three surveys for all plants and compounds addressed within BACCHUS were uploaded into the eBASIS-Creme Global exposure interface. This cloud-based tool was developed during the BACCHUS project and is enabled by a platform for collaboratively managing research data, hosting, and presenting user interfaces for these models [41].

## 3. Results

### 3.1. eBASIS Database Contents

Table 2 shows the contents of the entire eBASIS database and the data added during the course of BACCHUS (2012–2016). eBASIS has been extended with the total contents, including the composition of 794 bioactive compounds in 266 foods from 1147 quality-evaluated peer-reviewed publications, alongside data from 567 publications covering beneficial bioeffects carried out in human randomized controlled studies. The included data allow exposure assessment of bioactive compounds from foods to be estimated and modelled in human populations when used in conjunction with national food consumption data.

New bioactive compositional data has been added on 107 plants, with significant updates on cloudberry, aronia, strawberry, raspberry, orange, apple, and blackberry. A total of 231 references evaluated yielded over 10,000 records, each record is a unique entry for a specific plant/compound combination, and a single publication may yield from one to over 100 records. Table 3 summarizes eBASIS compositional data for the prioritized plants and includes the data from the seven fruits taken from 103 new peer-reviewed publications describing trials exploring their beneficial effects; in total, data included 17 plants.

With the eBASIS database adapted for bioactive peptide composition of meats and meat products, 38 papers covering meat from seven different animal types (turkey, chicken, beef, pork, lamb, rabbit, reindeer, and horse) were evaluated and data entry created 608 inputs on the composition of carnosine, taurine, citrulline, and anserine, which are detailed in Table 4.

### 3.2. Assessment of Bioactive Intakes

The eBASIS database export included 10,599 records, 86 plants, covering 242 individual compounds from seven compound classes: anthocyanins; ellagitannins and ellagic acid; flavanols; flavanones; flavones; flavonols; and pro(antho)cyanidins. Table 5 presents mean and 95th percentile (P95) results for daily intakes of selected foods and bioactives studied within the BACCHUS project and deemed to be major contributors of bioactive intakes. Standard errors for mean and percentiles, using a bootstrapping resampling technique, are also presented.

From the intake scenario (see Table 6) when using data from the human study, the mean daily epicatechin intake before the supplementation was 17.3 mg/day in UK adults, and these results apply to all consumers, including people that do not consume supplements. After the incorporation of the epicatechin capsules at 70 and 140 mg/capsule into the diet, the mean daily epicatechin intake increased to 35.4 mg/day and 53.5 mg/day, respectively, when looking at 100% consumption probability.

The development of the cloud-based eBASIS-Creme Global Exposure tool [41] allows viewing and filtering of database results based on user inputs. Summary statistics on bioactive compound intakes, from selected foods across the four populations, comprise of the average (mean), 95th percentile (P95), minimum, and maximum. The four populations being UK, Ireland, Spain and Norway, the Norwegian data has been included in the analysis for this manuscript due to data incompleteness, in Norway the data includes intakes of consumers only, not total population). After login to the exposure tool, the user first selects the country of interest from a drop-down list. The intake statistic, the compound, and the food of interest can then be specified. These statistics relate to the consumer type (average, high, minimum, or maximum) for which intakes will be displayed. The drop down list will only indicate possible options of compound food combinations. Once the query is submitted, the intakes of a specified compound for the chosen country in the chosen food will be returned, including an output table and a brief description.

## 4. Discussion

eBASIS continues to be one of the major databases on composition and beneficial bioeffects of bioactive compounds in foods, with all of the data traceable to original peer-reviewed publications. The inclusion of information on plant varieties, animal breeds, food processing, analytical methods, and transparent quality systems makes it an important, reliable resource for research.

The database has been extended by 27% to include the composition of 794 bioactive compounds in 267 foods from 1147 quality-evaluated peer-reviewed publications, alongside data from 567 publications covering beneficial bioeffects studies carried out in human randomized controlled trials. In both compositional and bioeffects sections a total of more than 40,000 records have been added, increasing the value of the database for researchers in the area of bioactive composition and biological effects.

Other bioactive compound composition databases have been developed in recent years. The USDA flavonoid database [38] was recently extended by combining their flavonoid, isoflavone, and proanthocyanidin databases, with additional methods used to assign logical zeros, moisture adjustments, and multi-ingredient foods. Phenol-Explorer, an open access database used widely for bioactive intake estimations, has had several major updates [42,43] and now includes new information on food processing, retention factors, and integration with human metabolome databases for chemical structures and molecular weights. Phenol-Explorer includes summary statistics, whereas eBASIS covers raw data, enabling the user to choose their preferred methods of aggregation and statistical analysis of bioactive composition and bioeffects.

Peterson et al. [21] carried out a detailed comparison in the characteristics of the three databases: USDA, Phenol-Explorer, and eBASIS. The values are generally similar because they are based largely on a common group of analytical data. eBASIS holds the greater number of compound classes and peer reviewed publications included. As is the nature of all databases, constant updating with new data is essential to keep the data relevant. The commonly used bioactive databases are falling behind with updates. USDA, currently on version 3.2, last had a major update in 2010 and Phenol-Explorer carried out their systematic reviews between 2005 and 2009. Table 3 of this manuscript highlights the quantity of data required to update new bioactive composition data in just 12 plants published in the last 10 years, leading to eBASIS being the most complete source for these foods. It must be noted, however, that all databases are unlikely to be complete for all compound/food combinations, with data on non-plant foods being much more incomplete. Researchers may find using a collection of databases a relevant way of ensuring they reach all data available where possible.

Peterson et al. [21] have compared bioactive composition databases for estimation of flavonoid intake in the study of health outcomes, and highlighted the challenges of incompleteness and the need for inclusion of data on plant varieties and herbs and spices, which are good sources of flavonoids. Scalbert et al. [44] highlighted important issues in the development of databases on food phytochemicals, including queryable, user-friendly systems, which are fully referenced and expandable. The updates in eBASIS have addressed many of these issues, with the addition of bioactive composition data on fruits previously not included and new published data not currently present in other databases, allowing the estimation of flavonoid intakes. All data within eBASIS is fully referenced and retrievable. The ability for eBASIS users to search for data from human intervention studies enhances its use for researchers and industry above that of databases used solely for compositional data.

There are few studies available on bioactive intakes in a population [39,40]. In one example, when comparing intakes to other published studies Grosso et al., 2014 [39] used compositional data from Phenol-Explorer [23] and reported mean hesperidin intakes from orange juice of 16.5 mg/day. As a comparison, the mean hesperidin concentration of orange juice observed here using the eBASIS data was 640 mg/kg, whilst in Phenol-Explorer the concentration lists a mean of 258 mg/kg.

The mean epicatechin concentration of apples using eBASIS data was 147 mg/kg and in Phenol-Explorer this was 287 mg/kg. Grosso et al. reported a mean daily epicatechin intake of approximately 16 mg/day from apples, which is higher compared to the intakes in the BACCHUS study (between 3 and 6 mg/day). When linking epicatechin intakes from apples to the epicatechin concentration in apples from Phenol-Explorer, the apple consumption described by Grosso et al. was 56 g/day. In comparison, mean daily apple intake in UK adults was 21 g/day and 45 g/day for apple consumers, both statistics including apple consumption from various dishes.

The Grosso et al. [39] study found the mean daily epicatechin intake from chocolate to be 6.4 mg/day, this compares to 3.4 to 6.7 mg/day in this eBASIS study, with the average epicatechin concentration of chocolate from eBASIS of 898 mg/kg whilst, in Phenol-Explorer, the concentration lists a mean of 704 mg/kg.

Dietary bioactive intake assessments are carried out as part of population health studies [39,40,45,46], with Peterson reviewing a number of intake studies reported in recent European, UK, US, and Australian cross-sectional and cohort studies [21]. In many cases, the results were limited due to recall bias and because they did not use representative intake data. However, the new interface linking eBASIS composition data with the Creme Global Exposure tool uses secondary data from national food consumption surveys to assess bioactive intake distributions in a population.

National food consumption surveys are used to assess and monitor dietary habits, food and nutrient intakes, as well as the health status in a population; however, intakes of bioactive compounds are not routinely assessed during these surveys. Few databases in Europe and the United States report bioactive compound intakes [21]. eBASIS was used to provide data for dietary intake assessments, as part of BACCHUS. It is notable that the three countries considered differed in their survey methodology in how dietary intakes are assessed and, hence, are not directly comparable. 

Overall, bioactive intakes were comparable to other studies, keeping in mind the differences in the population and dietary intake methodology, as well as bioactive concentration data. Differences in intakes were mainly due to food consumption being different. Additionally, databases, such as Phenol-Explorer, used in other studies capture mean concentrations resulting from a review of multiple studies. In contrast, this project focused on using a data distribution from eBASIS. An advantage of the intake analysis carried out within BACCHUS is that variability of some bioactive concentrations is accounted for [47]. such variability

According to the USDA databases of flavonoids and the Phenol-Explorer database, the mean daily intake of flavanols from apples was (10.6 mg/day and 28 mg/day, respectively) [40] compared to the intakes within the BACCHUS project (16.2 mg/day in UK adults). When combining the data on apple epicatechin concentration and intakes the daily apple consumption is derived as 56 g/day in Grosso et al. [39], which was higher than in the consumption surveys used in BACCHUS. The Grosso study covers intake of foods in a Polish cohort, based on a three-month food frequency questionnaire. The national food consumption data used within our study were deemed to be representative of a population’s diet, which makes the intake analysis more robust. Additionally, in the case of apples, the Phenol-Explorer data covers cider apples, where eBASIS contains data for all varieties.

When applying data from the BACCHUS human study, epicatechin intakes via supplementation with capsules were almost doubled and tripled in UK adults when given at 70 mg/day and 140 mg/day, showing the impact on dietary intakes via new products. Supplement consumers were deemed to have a higher acceptance for consuming bioactive capsules; hence, epicatechin capsules were given to supplement consumers only in the case study. It should be noted that total dietary epicatechin intakes only represent the foods analysed in eBASIS, which did not include bioactives from any other plant-based supplements.

The European Commission’s (EC) Nutrition and Health Claims Regulation (Regulation No. 1924/2006; EC 2007) came into force in 2007. Before this time, there was no regulatory control of health claims made about foods, beverages, or dietary supplements sold in Europe. Scientific assessment of evidence in support of health claims is carried out by EFSA, with the EC using EFSA’s scientific opinion (positive or negative) as evidence for a specific claim and to determine whether to approve or reject the claim [48]. For health claims, a dossier in support of the application must be prepared and include characterisation of the active substance and bioavailability of the food/constituent. Bioactive composition databases and information on bioactive intakes within a normal diet may be favourable for a dossier.

eBASIS contains compositional data which may be used for intake estimation, and uniquely, it also contains quality-evaluated bioeffects information from peer reviewed publications, and this has been extended to contain 1117 records from 567 studies providing data on biomarkers mainly relating to cardio-metabolic and bone health outcomes. Kiely et al. [25] have shown there is extensive overlap between eBASIS and the EU-submitted health claims that relate to plant-based bioactive compounds, confirming eBASIS is a useful tool for regulators to independently check completeness of health claim applications relating to phytochemicals, and is a potentially valuable resource to assist claimants in the compilation of dossiers on functional foods and health.

The newly-extended eBASIS database and its link to a probabilistic intake model are included within an online toolbox developed during the BACCHUS project, to help food businesses, especially SMEs, and researchers submit better health claim dossiers [48]. As part of the toolbox, a user interface was developed, using the resulting output data from the bioactive intake model. The tool primarily aims to help SMEs determine whether the quantities of foods or compounds required for the claimed effect are consumed at present and, thus, it can be assumed that these intake levels are realistically achievable within current dietary habits; a requirement for a health claim [49]. Other questions may also be answered using this tool. The interface forms a part of the BACCHUS toolkit, an interactive platform with five integrated tools and resources aimed at SMEs, researchers, and regulators, including e-learning and a best practice guide to making health claims.

## Figures and Tables

**Table 1:** Categories of data in the eBASIS database export used within probabilistic intake model to assess bioactive intakes.

Plant Names			Compound Classes	Selected Output Fields
Almond Apple Apple Juice Apricot Aubergine, eggplant Avocado Banana Bean, Faba Bean, kidney Blackberry Blackcurrant Blackcurrant Juice Blueberry Broccoli Cabbage, Chinese Cabbage, red Cabbage, white Carrot Celeriac Cherry Chicory Chocolate Chocolate drink Clementine mandarin Coriander Cranberry Juice Cucumber	Dill Elder Juice Fennel Fig Garlic Ginkgo product Gooseberry Grape (fruit) Grape (fruit) Juice Grape (Wine) Grapefruit Grapefruit Juice Green tea dietary supplement Guava Kiwi Leek Lemon Lemon Juice Lettuce Lime Lime Juice Mandarin Mango Nectarine Onion Orange Juice Orange, Seville	Orange, sweet Papaya Parsley Pea, garden Peach Pear Pecan Pepper, bell Persimmon Pineapple Pistachio Plum Pomegranate Pomegranate Juice Pomelo Potato Radish, Japanese Raspberry Rice Rosemary Sage Soya Spinach Strawberry Strawberry Juice Sweet potato Tea Tomato Walnut Watercress Watermelon	Anthocyanins Flavanols Ellagitannins and Ellagic Acids Flavanones Flavonols Pro(antho)cyanidins Flavanols	Scientific name Compound Level Unit Part Plant description Shape, state or form EuroFIR classification Heat treatment Cooking method Treatment applied Preservation method Quality code

**Table 2:** The entire content of eBASIS and new data added during BACCHUS project.

Data Type	Plants	Compounds	References	Records
**Composition**				
Total	267	794	1147	39,756
Via BACCHUS	107	339	231	10,679
**Bioeffects**				
Total	88	168	567	1117
Via BACCHUS	17	19	103	106

**Table 3:** The entire eBASIS data on composition of bioactives in selected fruits and nuts.

Fruit	Composition Data			Beneficial Bioeffects Data		
	No. Inputs	No. Compounds	No. Refs	No. Inputs	No. Compounds	No. Refs
Apple	2329	95	70	13	7	13
Blackberry	460	92	33	2	2	2
Cacao	132	32	14	28	8	22
Chokeberry	491	53	40	10	5	10
Cloudberry	70	42	12	2	2	2
Mandarin	154	34	24	3	3	2
Orange, Seville	316	47	25	1	1	1
Orange, sweet	1032	114	75	6	3	6
Pomegranate	1112	73	29	27	6	25
Raspberry	1087	143	55	4	3	4
Strawberry	2380	141	70	14	5	14
Walnut	105	34	13	23	2	23

**Table 4:** eBASIS data on the composition of bioactive compounds in meats and meat products.

Animal	No. Inputs	No. Compounds	No. Refs
Bovine (beef and other bovine animals/meats, e.g., ox, buffalo)	111	4	11
Equine (horse and other equine animals/meats)	2	2	1
Leporine (rabbit or hare)	2	2	1
Other game mammals (e.g., moose, reindeer)	18	3	2
Ovine (lamb, mutton and other ovine animals/meats)	22	2	1
Porcine (pork, ham, bacon and other porcine animals/meats, e.g., wild boar)	77	3	9
Poultry (e.g., chicken, turkey)	376	3	20

**Table 5:** Mean (mean standard error, SE) and P95 (P95 standard error, SE) food (g/day) and compound intakes (mg/day) in three European countries.

Food and Compound	Ireland				UK				Spain			
	Mean	SE	P95	SE	Mean	SE	P95	SE	Mean	SE	P95	SE
Apple intake g/day	28	1.2	120	4.9	21	0.9	88	5.7	40	1.1	169	11
*Cyanidin-3-galactoside (Anthocyanins)*	0.66	0.1	4.48	0.3	0.55	0	3.83	0.1	0.91	0	5.16	0.9
*Epicatechin (Flavanols)*	3.96	0.2	20.7	1.4	3.16	0.2	15.3	0.96	6.06	0.2	30.7	1.3
*Procyanidin (Pro(antho)cyanidins)*	2.52	0.1	11.8	0.53	2.01	0.1	8.83	0.47	3.8	0.1	18.1	0.4
*Quercetin glycosides (Flavonols)*	11.58	1.1	118	14.3	9.35	0.9	112	16.7	16.86	1.2	151	51
Orange juice intake g/day	29.89	1.7	165	8.7	35	2	200	14	35.36	1.4	200	0
*Cyanidin-3-glucoside (Anthocyanins)*	0.96	0.1	5.86	0.33	1.06	0.1	6.58	0.39	1.17	0.1	7.73	0.3
*Epicatechin (Flavanols)*	0.9	0.1	5.16	0.25	1.07	0.1	5.47	0.4	1.08	0	6.08	0.3
*Hesperidin (Flavanones)*	10.2	0.7	59.2	3.43	11.75	0.9	65.4	4.85	12.33	0.6	66.7	3
*Neohesperidin (Flavanones)*	0.67	0.1	2.79	1.39	0.79	0.1	3.92	1.58	0.81	0.1	1.99	0.4
Tea intake g/day	446	11	1130	31.1	438	10	1200	27.1	20.19	1.4	133	6.5
*Catechin gallate (Flavanols)*	310	7.7	825	29	309.3	7.8	924	19.1	14.9	1.2	93.6	5.8
*Epicatechin (Flavanols)*	12.96	0.3	35.6	1.5	12.9	0.3	38.2	0.96	0.6	0.05	4.7	0.4
*Kaempferol glycosides (Flavonols)*	18.8	0.5	57	2.04	18	0.5	57.8	1.39	0.87	0.1	2.9	0.6
*Procyanidin B1 (Pro(antho)cyanidins)*	6.78	0.2	18	0.71	6.7	0.2	19	0.4	0.31	0	2.1	0.2
Chocolate intake g/day	3.38	0.2	18.5	1.8	7.6	0.4	35	1.7	4.27	0.2	23.2	1.4
*Epicatechin (Flavanols)*	3.36	0.3	23.8	1.21	6.71	0.4	35.5	2.4	3.88	0.3	22.6	1.8
*Catechin (Flavanols)*	0.88	0.1	5.01	0.52	1.85	0.1	10.1	0.5	1.18	0.1	7.02	0.4
*Procyanidin B2 (Pro(antho)cyanidins)*	2.11	0.2	13.3	0.8	4.58	0.3	24.4	1.39	2.7	0.2	14.8	1.1
*Procyanidin polymers (Pro(antho)cyanidins)*	14.72	1	91.9	6.99	34.66	1.8	170	6.4	19.3	1.2	109	8.2

**Table 6:** Dietary epicatechin intake in UK adults when incorporating epicatechin capsules at two different doses (70 and 140 mg/capsule) at various probabilities of consumption.

Epicatechin Intakes (mg/Day), *N* = 2083									
Intake Scenario	Probability of Consumption	Mean	SE	P25	SE	Median	SE	P95	SE
Baseline Diet	-	17.3	0.4	5.1	0.4	14.7	0.5	44.8	0.7
Plus Epicatechin Capsule 70 mg	0.5	27	0.6	7.2	0.5	19.5	0.7	75.5	1.6
	0.75	30.8	0.7	7.1	0.5	19.9	0.7	89.2	1.6
	1	35.4	0.8	7.2	0.5	20.7	0.7	103.2	1.4
Plus Epicatechin Capsule 140 mg	0.5	36.1	1	6.9	0.6	20.2	0.9	122.7	2.3
	0.75	44.6	1.2	7.1	0.6	20.6	0.9	154.9	1.7
	1	53.5	1.5	7.4	0.6	20.9	0.8	173.8	1.3

## Supplementary Materials

The following are available online at http://www.mdpi.com/2072-6643/9/4/320/s1. Table S1: New eBASIS fields required for meat inputs.
