# Supplementary material for: eBASIS (Bioactive Substances in Food Information Systems) and Bioactive Intakes: Major Updates of the Bioactive Compound Composition and Beneficial Bioeffects Database and the Development of a Probabilistic Model to Assess Intakes in Europe
Source: Nutrients. 2017 Mar 23;9(4):320. doi: 10.3390/nu9040320 (PMC5409659; doi:10.3390/nu9040320)
Supplement: Supplementary file 1 [file nutrients-09-00320-s001.docx]

Supplementary Material

eBASIS (Bioactive Substances in Food Information Systems) and Bioactive Intakes: Major updates of the Bioactive Compound Composition and Beneficial Bioeffects Database and the Development of a Probabilistic Model to Assess Intakes in Europe

Jenny Plumb , Sandrine Pigat, Foteini Bompola, Maeve Cushen, Hannah Pinchen, Eric Nørby, Siân Astley, Jacqueline Lyons, Mairead Kiely and Paul Finglas

**Supplementary Table S1.** New eBASIS fields required for meat inputs.

| **Plant Field** | **New Meat Field** | **Description** |
| --- | --- | --- |
| Plant | Meat or animal | Meats grouped according to animal groups, such as bovine, porcine, ovine etc. (pick list) |
| Part | Muscle or cut | Rather than attempt to characterize all possible muscle cuts, this is not a pick list and differentiates between muscle and organs in a free-text field (e.g., heart, liver, cheek, rib etc.) |
| Sub-species/Cultivar | Breed or sub-species | Animal characteristics may be important to composition, therefore if the breed or sub-species is provided it is documented (free text) |
| Maturity | Sex, Maturity or age at slaughter | Since there is a significant variation in how animal maturity is reported this is a free-text field. The sex of the animal(s) should also be reported (free text) |
| Country of Origin | Country of origin | In the case of meat products, it must be clear whether the origin is the meat (country of rearing) or the product, as they may not be the same (pick list) |
| Region | Region | Region reared or region meat product produced (free text) |
| Season | Wild/reared | Define whether the meat is from a wild or reared animal (free text) |
| Growing conditions | Rearing conditions | Free text to allow description of livestock rearing conditions, if given |
| Generic food name | Generic food name | Particularly used for meat products, e.g., sausage, ham (free text) |
| Commercial food name | Commercial food name | Addition of commercial names (free text) |
| GMO |  | Not required |
| Diseased plant |  | Not required |
| Plant description | Meat/product description | A full description of the meat, cut, or meat product |
